# Detection of ERG11 point mutations in Iranian fluconazole-resistant *Candida albicans* isolates 

**DOI:** 10.18502/cmm.5.1.531

**Published:** 2019-03

**Authors:** Ali Sardari, Hossein Zarrinfar, Rasoul Mohammadi

**Affiliations:** 1 Department of Medical Parasitology and Mycology, School of Medicine, Isfahan University of Medical Sciences, Isfahan, Iran; 2 Allergy Research Center, Mashhad University of Medical Sciences, Mashhad, Iran; 3 Department of Medical Parasitology and Mycology, School of Medicine, Infectious Diseases and Tropical Medicine Research Center, Isfahan University of Medical Sciences, Isfahan, Iran

**Keywords:** Candida albicans, ERG11 gene, Fluconazole, Minimum inhibitory concentration

## Abstract

**Background and Purpose:**

Candidiasis is referred to a group of superficial and deep-tissue fungal infections often caused by *Candida albicans. *The superficial infections affect the oral, oropharynx, esophagus, and vaginal mucosa*. *The treatment of choice for these infections is the use of azoles, such as fluconazole. However, the increased use of these antifungal agents has led to the emergence of azole-resistant isolates of *C. albicans*. Different mechanisms have been suggested for the development of drug resistance, such as mutations in the encoding gene *ERG11*. Mutations in *ERG11 *result in changes in the ERG11p spatial construction and reduce the affinity between the protein and azole. This study aimed to determine the susceptibility profile of *C. albicans* clinical isolates to fluconazole using microdilution method. The present research was also targeted toward the detection of mutations that might be related to fluconazole resistance by the amplification and sequencing of *ERG*11 gene.

**Materials and Methods:**

This study was conducted on a total of 216 clinical isolates obtained from Mashhad, Isfahan, and Tehran cities in Iran, during 2016-2018. The clinical isolates were identified using molecular techniques. Furthermore, minimum inhibitory concentration (MICs) was determined according to the clinical and laboratory standards institute M27-A3 and M27-S4 documents. The concentration range for fluconazole was obtained as 0.063-64 μg/ml. In the resistant strains, *ERG*11 genes were amplified by specific primers. Subsequently, cycle sequencing reactions were performed on purified polymerase chain reaction (PCR) products in forward and reverse directions. Finally, the results were analyzed by MEGA (version 7) and Gene Runner software (version 6.5.30).

**Results:**

Out of 216 strains, 100 (46.3%) species were identified as *C. albicans*. The MIC values for fluconazole had a range of 0.125-16 μg/ml with the MIC50 and MIC90 values of 0.5 and 1 μg/ml, respectively. Totally, 41 nucleotide changes were detected among 4 resistant isolates. In this regard, 4 out of 41 mutations in codons caused changes in ERG11p; however, these mutations did not lead to fluconazole resistance.

**Conclusion:**

Fluconazole resistance among clinical isolates is not merely due to the changes in ERG11p. This resistance may be also related to some other mechanisms, such as the prevention of the intracellular accumulation of the antifungal agent and alteration of the target enzyme to diminish drug binding.

## Introduction

*Candida albicans *is an important opportunistic yeast that can cause mucosal infections, such as thrush and oropharyngeal candidiasis (OPC) in AIDS patients, as well as systemic life-threatening infections in immunocompromised patients [1]. The use of potent antifungal agents is crucial for the management of these potentially fatal infections. Azole antifungal agents, such as fluconazole, are the treatment of choice for these infections [2]. 

However, the increased use of these antifungal agents, especially in HIV/AIDS patients receiving long-term therapy, has resulted in the emergence of azole-resistant isolates of *C. albicans*. Azole resistance mechanisms are different and may contribute to mutations in encoding gene *ERG11*. It is based on the spatial configuration alterations of the target enzyme 14a-demethylase (ERG11p) [3, 4]. Erg11p is a significant protein of the cytochrome P450 superfamily enzyme and has an important role in the ergosterol synthesis pathway of *C. albicans*. Ergosterol (ergosta-5,7,22-trien-3β-ol) is a kind of sterol found in the fungal cell membranes and play a major role in maintaining the integrity and function of *C. albicans* membrane [5]. 

Mutations in *ERG*11 result in changes in the ERG11p spatial construction and reduce the affinity between the protein and azole [6]. The aim of the present study was to determine the susceptibility pattern of *C. albicans* clinical isolates to fluconazole using microdilution method. This study was also targeted toward the detection of mutations that might be responsible for fluconazole resistance by the amplification and sequencing of *ERG*11 gene.

## Materials and Methods

***Clinical Isolates***

This study was conducted on a total of 216 clinical isolates obtained from Tehran (n=67), Isfahan (n=37), and Mashhad (n=112) cities in Iran, from July 2016 to February 2018. All strains were sub-cultured on Sabouraud glucose agar (SGA; Difco, Detroit, MI, USA) and incubated at 37°C for 24-48 h. 

***Molecular Identification***

***DNA Extraction***

Genomic DNA was extracted using the boiling method. Briefly, a bit of fresh colonies were suspended in 80 µL of distilled water and boiled for 20 min. They were then centrifuged for 5 min at 6,000 rpm. The resultant supernatant was used for polymerase chain reaction (PCR). Clinical isolates of Tehran and Isfahan were identified using the PCR-restriction fragment length polymorphism (PCR-RFLP) of the ITS1-5.8S-ITS2 regions as described previously [7, 8]. Furthermore, the identification of the clinical samples of Mashhad was performed through matrix-assisted laser desorption/ionization-time of flight (MALDI-TOF or MT-MS) mass spectrometry (Bruker Daltonics, Bremen, Germany), which is a new method. 

***In vitro antifungal susceptibility testing***

Minimum inhibitory concentration (MICs) was determined according to the clinical and laboratory standards institute M27-A3 and M27-S4 documents [9, 10]. Antifungal effect of fluconazole (Pfizer Central Research, Sandwich, United Kingdom) was evaluated on the clinical isolates of *C. albicans*. To this end, fluconazole was diluted in the RPMI-1640 medium (Sigma Chemical Co.), buffered to pH 7.0 with 0.165 M morpholinepropanesulfonic acid (MOPS; Sigma) with L-glutamine without bicarbonate. 

A final concentration of 0.063-64 μg/ml was considered for fluconazole. The MIC results were read after 24 h of incubation at 35°C. These values were determined visually as the lowest concentration of drug that caused a significant (>50%) reduction in the microorganism growth. According to the M27-S4 document, a fluconazole concentration of > 8 was considered for the resistant isolates [10].

***Amplification of ERG11 gene and sequencing***

Three pairs of primers were used for the amplification of *ERG11* gene [6]. ERGSec1A (50-TTAGTGTTTTATTGGATTCCTTGGTT-30) and ERGSec1B (50-TCTCATTTCATCACCAAATAAAGATC-30) yielded an amplicon expanding from 295 to 777 bp of the *ERG11* gene. Furthermore, ERGSec2A (50-ACCAGAAATTACTATTTTCACTGCTTCA-30) and ERGSec2B (50-AAGTCAAATCATTCAAATCACCA CCT-30) yielded a product extending from 723-1204 bp of the *ERG11* gene. Finally, ERGSec3A (50-AGGTGGTGATTTGAATGATTTGACTT-30) and ERGSec3B (50-GAACTATAATCAGGGTCAGGCACTTT-30) provided an expected PCR product extending from 1179-1667 bp of the *ERG11* gene. 

One susceptible isolate (No. 27) was used as the control strain. The PCR mixture included 2.5 μl of 10X reaction buffer, 0.5 ml of 30 pmol/ml of each primer, 0.5 ml of 10 mM dNTP, 0.25 ml of Taq polymerase (5U/ml), and 2 μl of extracted DNA in a final volume of 25 μl. The PCR products were purified by means of the ethanol purification method. The cycle sequencing reactions were performed in forward and reverse directions (Bioneer, Korea). 

The sequencing products were aligned and analyzed in comparison with the nucleotide sequence of cytochrome P450 L1A1 (lanosterol 14 alpha-demethylase) from *C. albican *[11] and published *ERG*11 sequence in GenBank by Lai and Kirsch (accession number: X13296) using MEGA (version 7) and Gene Runner software.

## Results

Out of 216 strains, 100 (46.3%) species were identified as *C. albicans*. The majority of the patients (23%) were in the age group of 31-40 years. Most of the specimens were collected from vaginitis (30%), onychomycosis (26%), and bronchoalveolar lavage (BAL; 21%) cases. The study population had a male to female ratio of 31:69. The most frequent predisposing factors among the patients included pregnancy (29%), cancer (9%), and diabetes mellitus (9%). An MIC range of 0.125-16 μg/ml was obtained for fluconazole having the MIC50 and MIC90 values of 0.5 and 1 μg/ml, respectively. 

Table 1 summarizes the demographic and clinical data of the patients enrolled in the present study. Isolates number 3, 11, 15, 23, and 45 were resistant to fluconazole (MIC=16); therefore, they were applied for sequencing. However, isolate number 3 was contaminated with *Aspergillus* species and did not recover on SGA. Totally, 41 nucleotides changes were detected among the isolates. Out of 41 mutations, 4 mutations in codons caused changes in ERG11p (Table 1) [6, 12-17]. 

**Table 1:** Characteristics of patients with candidiasis and nucleotide changes and mutations of *Candida albicans* *ERG11* gene and correlated changes in ERG11p among resistant isolates in the present study

**No**	**City**	**Age**	**Gender**	**Source of specimen**	**Risk factor**	**MIC (µg/ml)**	**Site of nucleotide (bp) mutation**	**Nucleic acid mutation**	**Amino acid substitution**	**Change in** **ERG11p**
1	Mashhad	80	F	BAL	-	0.125	-	-	-	-
2	Mashhad	39	F	Vaginitis	Pregnancy	0.25	-	-	-	-
3	Mashhad	55	M	BAL	Hepatitis	16	-	-	-	-
4	Mashhad	39	F	Bladder Biopsy	-	0.25	-	-	-	-
5	Mashhad	65	M	BAL	Lung Cancer	0.25	-	-	-	-
6	Mashhad	52	M	BAL	-	0.125	-	-	-	-
7	Mashhad	42	F	Vaginitis	Pregnancy	0.125	-	-	-	-
8	Mashhad	39	F	Vaginitis	Pregnancy	0.25	-	-	-	-
9	Mashhad	28	F	Vaginitis	Pregnancy	0.25	-	-	-	-
10	Mashhad	48	M	BAL	-	0.125	-	-	-	-
11	Mashhad	28	F	Vaginitis	Pregnancy	16	462 558 696 805 1143	TTT→TTC TCC→TCT CAT→CAC CTA→TTA GTT→GTC	Phe→Phe Ser→Ser His→His Leu→Leu Val→Val	N/C N/C N/C N/C N/C
12	Mashhad	55	F	BAL	Lung cancer	0.125	-	-	-	-
13	Mashhad	63	M	BAL	Leukemia	0.25	-	-	-	-
14	Mashhad	44	M	BAL	Lung cancer	4	-	-	-	-
15	Mashhad	45	M	Joint	Rheumatic diseases	16	462 504 558 805 945 1143 1167 1257 1350 1443 1449 1587 1609 1617	TTT→TTC AAA→AAG TCC→TCT CTA→TTA GAA→GAC GTT→GTC TTA→TTG CTC→CTT TAT→TAC GCC→GCT GCT→GCC TTA→TTG GTT→ATT AAT→AAC	Phe→Phe Lys→Lys Ser→Ser Leu→Leu Glu→Asp Val→Val Leu→Leu Leu→Leu Tyr→Tyr Ala→Ala Ala→Ala Leu→Leu Val→Ile Asn→Asn	N/C N/C N/C N/C E266D^12^ N/C N/C N/C N/C N/C N/C N/C V488I^12^ N/C
16	Mashhad	65	M	BAL	Lung cancer	0.25	-	-	-	-
17	Mashhad	79	F	BAL	-	0.25	-	-	-	-
18	Mashhad	45	F	BAL	Lung cancer	0.25	-	-	-	-
19	Mashhad	29	F	Vaginitis	Pregnancy	0.25	-	-	-	-
20	Mashhad	39	M	BAL	Kidney transplantation	0.25	-	-	-	-
21	Mashhad	53	F	Urine	Kidney transplantation	0.25	-	-	-	-
22	Mashhad	53	M	BAL	-	0.125	-	-	-	-
23	Mashhad	74	F	BAL	Lung Cancer	16	696 805 1143 1287 1350 1443 1449	CAT→CAC CTA→TTA GTT→GTC TTT→TTC TAT→TAC GCC→GCT GCT→GCC	His→His Leu→Leu Val→Val Phe→Phe Tyr→Tyr Ala→Ala Ala→Ala	N/C N/C N/C N/C N/C N/C N/C
24	Mashhad	70	F	Biopsy	Diabetes mellitus	0.25	-	-	-	-
25	Mashhad	35	F	Vaginitis	Pregnancy	0.5	-	-	-	-
26	Mashhad	45	F	Vaginitis	Pregnancy	0.25	-	-	-	-
27	Mashhad	29	F	Vaginitis	Pregnancy	0.125	-	-	-	-
28	Mashhad	44	F	Vaginitis	Pregnancy	0.5	-	-	-	-
29	Mashhad	29	F	Vaginitis	Pregnancy	0.5	-	-	-	-
30	Mashhad	35	F	Thrush	Lupus	0.25	-	-	-	-
31	Mashhad	40	M	BAL	Rheumatic diseases	0.25	-	-	-	-
32	Mashhad	28	F	Vaginitis	Pregnancy	0.125	-	-	-	-
33	Mashhad	33	F	Vaginitis	Pregnancy	0.125	-	-	-	-
34	Mashhad	26	F	Vaginitis	Pregnancy	0.5	-	-	-	-
35	Mashhad	21	F	Vaginitis	Pregnancy	0.125	-	-	-	-
36	Mashhad	32	F	Vaginitis	Pregnancy	0.25	-	-	-	-
37	Mashhad	22	F	Vaginitis	Pregnancy	0.25	-	-	-	-
38	Mashhad	31	F	Vaginitis	Pregnancy	0.5	-	-	-	-
39	Mashhad	43	F	Vaginitis	Pregnancy	0.25	-	-	-	-
40	Mashhad	22	F	Vaginitis	Pregnancy	0.25	-	-	-	-
41	Mashhad	24	F	Vaginitis	Pregnancy	0.125	-	-	-	-
**Table 1. **Continued										
42	Mashhad	29	F	Vaginitis	Pregnancy	0.125	-	-	-	-
43	Mashhad	28	F	Vaginitis	Pregnancy	0.125	-	-	-	-
44	Mashhad	36	F	Vaginitis	Pregnancy	0.5	-	-	-	-
45	Mashhad	28	F	Vaginitis	Pregnancy	16	462 504 558 696 805 945 1143 1167 1257 1350 1443 1449 1587 1609 1617	TTT→TTC AAA→AAG TCC→TCT CAT→CAC CTA→TTA GAA→GAC GTT→GTC TTA→TTG CTC→CTT TAT→TAC GCC→GCT GCT→GCC TTA→TTG GTT→ATT AAT→AAC	Phe→Phe Lys→Lys Ser→Ser His→His Leu→Leu Glu→Asp Val→Val Leu→Leu Leu→Leu Tyr→Tyr Ala→Ala Ala→Ala Leu→Leu Val→Ile Asn→Asn	N/C N/C N/C N/C N/C E266D^12^ N/C N/C N/C N/C N/C N/C N/C V488I^12^ N/C
46	Mashhad	44	F	Vaginitis	Pregnancy	0.5	-	-	-	-
47	Mashhad	73	M	BAL	Lung cancer	0.25	-	-	-	-
48	Mashhad	34	F	Vaginitis	Pregnancy	0.5	-	-	-	-
49	Mashhad	26	F	Vaginitis	Pregnancy	0.5	-	-	-	-
50	Mashhad	32	F	Vaginitis	Pregnancy	0.25	-	-	-	-
51	Mashhad	64	M	Urine	Kidney transplantation	0.5	-	-	-	-
52	Isfahan	24	M	Groin	-	0.5	-	-	-	-
53	Isfahan	28	F	Fingernail	-	0.5	-	-	-	-
54	Isfahan	37	F	Toenail	-	0.25	-	-	-	-
55	Isfahan	16	F	Fingernail	Diabetes mellitus	0.25	-	-	-	-
56	Isfahan	35	M	Fingernail	-	0.5	-	-	-	-
57	Isfahan	39	M	Skin	-	2	-	-	-	-
58	Isfahan	48	F	Fingernail	-	0.5	-	-	-	-
59	Isfahan	69	F	Fingernail	-	0.5	-	-	-	-
60	Isfahan	41	M	BAL	-	0.5	-	-	-	-
61	Isfahan	39	F	Fingernail	-	0.5	-	-	-	-
62	Isfahan	3	M	Thrush	-	0.5	-	-	-	-
63	Isfahan	40	F	Vaginitis	-	0.5	-	-	-	-
64	Isfahan	29	F	Fingernail	Diabetes mellitus	1	-	-	-	-
65	Isfahan	14	F	Groin	-	2	-	-	-	-
66	Isfahan	45	F	Fingernail	-	2	-	-	-	-
67	Isfahan	56	F	Fingernail	-	0.5	-	-	-	-
68	Isfahan	39	M	Blood	Leukemia	1	-	-	-	-
69	Isfahan	19	M	Toenail	-	0.5	-	-	-	-
70	Tehran	74	F	Groin	Diabetes mellitus	0.25	-	-	-	-
71	Tehran	65	F	BAL	-	0.5	-	-	-	-
72	Tehran	71	F	Groin	-	0.5	-	-	-	-
73	Tehran	78	M	Sputum	-	2	-	-	-	-
74	Tehran	48	M	Lung Biopsy	-	0.5	-	-	-	-
75	Tehran	68	F	Toenail	-	0.5	-	-	-	-
76	Tehran	52	F	BAL	-	0.5	-	-	-	-
77	Tehran	39	F	Fingernail	Diabetes mellitus	0.5	-	-	-	-
78	Tehran	61	F	Fingernail	-	0.25	-	-	-	-
79	Tehran	40	F	Toenail	-	0.5	-	-	-	-
80	Tehran	65	M	Toenail	Diabetes mellitus	0.5	-	-	-	-
81	Tehran	70	M	Interdigitale	-	0.5	-	-	-	-
82	Tehran	28	F	Fingernail	-	0.25	-	-	-	-
83	Tehran	34	M	Toenail	Diabetes mellitus	0.5	-	-	-	-
84	Tehran	19	F	Fingernail	-	1	-	-	-	-
85	Tehran	48	F	Fingernail	-	0.5	-	-	-	-
86	Tehran	18	M	Cornea	-	0.5	-	-	-	-
87	Tehran	77	F	BAL	-	0.25	-	-	-	-
88	Tehran	61	M	Sputum	-	0.5	-	-	-	-
89	Tehran	59	F	Fingernail	-	1	-	-	-	-
90	Tehran	47	F	Fingernail	Diabetes mellitus	0.5	-	-	-	-
91	Tehran	62	F	Fingernail	-	0.25	-	-	-	-
92	Tehran	34	F	Groin	Hyperhidrosis	0.25	-	-	-	-
**Table 1. **Continued										
93	Tehran	46	F	Toenail	-	0.25	-	-	-	-
94	Tehran	58	M	Fingernail	-	0.25	-	-	-	-
95	Tehran	70	F	Toenail	-	0.5	-	-	-	-
96	Tehran	86	F	Groin	-	0.25	-	-	-	-
97	Tehran	27	M	Esophagitis	-	0.25	-	-	-	-
98	Tehran	10	M	Thrush	Diabetes mellitus	0.5	-	-	-	-
99	Tehran	65	F	BAL	-	0.5	-	-	-	-
100	Tehran	58	M	Skin	Hyperhidrosis	0.5	-	-	-	-

* BAL: Bronchoalveolar lavage, MIC: minimum inhibitory concentration, M: male, F: female, N/C: no change, aa: amino acid, Phe: phenylalanine, Ser: serine, His: histidine, Leu: leucine, Val: valine, Lys: lysine, Glu: glutamine, Asp: aspartic acid, Tyr: tyrosine, Ala: alanine, Ile: isoleucine, Asn: asparagine

## Discussion

Candidiasis is referred to both superficial and deep-tissue fungal infections often caused by *C. albicans*. Based on the epidemiological surveys performed in the Europe [18], United States [19], and the Middle East [20], the superficial types can affect the oral, oropharynx, esophagus, and vaginal mucosa. *Candida albicans* is the main cause of invasive *Candida* infections in the majority of obtained clinical specimens, accounting for 50-70% of the cases [21]. The treatments of *Candida* infections differ considerably and are based on the patients' underlying disease and immune status, anatomic location of the infection, *Candida* species responsible for infection, predisposing factors, and, in some cases, the susceptibility of *Candida* species to antifungal agents [22, 23]. 

Nystatin, itraconazole, miconazole, voriconazole, echinocandins, flucytosine, and amphotericin B are the anti-*Candida* agents applied for the management of this infection. However, the most prevalently recommended antifungal used for the majority of *C. albicans* isolates is fluconazole, which is a member of the azole class [24]. Azoles inhibit 14-α-sterol demethylase encoded by *ERG11* gene, which is a significant enzyme involved in the biosynthesis of ergosterol (i.e., fungal-specific membrane sterol).

Some investigations have shown the ability of *Candida* species to expand high resistance to azole agents [25, 26]. Prior ﬂuconazole exposure has been shown to increase the risk of ﬂuconazole resistance in *Candida* species [26]. Increased resistance to fluconazole is caused by different factors, such as the change of the target enzyme to diminish drug binding, reduction of the toxic effects of the antifungal, enhancement of the amount of the target enzyme, prevention from the intracellular accumulation of the antifungal agent [27], raised expression of *ERG11* as a result of activating mutations in the gene encoding the zinc-cluster transcriptional regulator Upc2p [28], overexpression of drug efflux pumps [29], and inactivation of *ERG3* gene [30]. 

*Candida albicans* strains isolated from patients with candidemia have the lowest incidence of azole resistance (0-5%) [31, 32]. However, the incidence of fluconazole resistance among the *C. albicans* clinical isolates of OPC is higher and is associated with prior OPC infections and previous fluconazole treatment [33]. Out of the 100 *C. albicans* clinical isolates, 5 isolates were resistant to fluconazole (MIC=16 μg/ml). Interestingly, all resistant strains belonged to Mashhad. 

Resistant isolates were obtained from BAL (n=2), vaginitis (n=2), and joint (n=1). One isolate (from BAL) did not grow after re-passaging on SGA; therefore, it was excluded from the study. Amino acid substitutions have been delineated in 61 parts of the ERG11p due to mutations in *ERG11* gene [6]. Erg11p mutations, such as D153E, E266D, D116E, K128T, K147R, E266Q, K287R, G129A, and G303D, do not cause fluconazole resistance [12-14, 34, 35]. However, ERG11p mutations, such as G464S, Y132H, S405F, R467K, T315A, and I471T, have been shown to cause resistance to azoles [34, 36-40]. 

The C-terminal and N-terminal of ERG11p are on the protein surface rather than on the active site; therefore, the 295-1667 bp part of *ERG11* gene was amplified to look for possible mutations. Biofilms, multidrug transporters in *Candida* species, multiple drug resistance gene *MDR1*, drug segregation within intracellular vacuoles, and also overexpression of *CDR1 *and* CDR2* as *Candida *drug-resistant genes can significantly reduce the intracellular drug concentration [4, 41, 42].

Susceptibility to fluconazole in *C.* *albicans* isolates may reach to 95.8% [43, 44]. However, as indicated in a number of investigations, the fluconazole resistance of such clinical isolates might be as high as 35% [45, 46]. In the present study, the incidence rate of fluconazole resistance in *C. albicans* was 5%. This low rate may be related to the administration of an appropriate dosage of antifungal agents or completion of the therapeutic course. 

In this study, two types of mutations, including E266D and V488I, were detected among the resistant isolates. E266D mutation was also reported previously; nonetheless, this mutation does not result in fluconazole resistance [12]. V488I (G1609A) mutation may not also lead to fluconazole resistance because V488 is located far from the active center of ERG11p [47]. Khademi et al. found four resistant *C. albicans* strains among patients in Guilan, Iran. Missense mutations, such as C470G, K291N, and Q474R, were detected in all resistant isolates. 

Similar to our results, Teymori et al. [48] detected 5 (5.1%) resistant *C. albicans* isolates (MIC≥64 μg/ml) out of 97 clinical samples. They revealed that *ERG*11 gene in the five fluconazole-resistant *C. albicans* isolates was up-regulated 4.15-5.84 folds, compared to the control strain. Eftekhari et al. [49] isolated 4 resistant *C. albicans* out of 40 strains and screened *ERG*11 gene mutations by PCR sequencing. Their isolated strains had D116E and V456G polymorphisms. 

In another study carried out during April 2015-April 2016, Alizadeh et al. [50] isolated 28 strains of *C. albicans* from muco-cutaneous candidiasis among immunocompromised patients in Omidiyeh, Khuzestan, Iran. In the mentioned study, all isolates were resistant to fluconazole. They evaluated *ERG* gene expression by semi-quantitative reverse transcriptase-(RT) PCR. In line with the results of the present study, they showed no significant changes in fluconazole-resistant isolates in comparison with untreated controls and *ERG*11 reference sequence. 

In 2017, Balabandi et al. [51] detected two missense mutations (i.e., D116E and E266D) in *ERG*11 gene among 20 resistant *C. albicans* in Rasht, Iran. Furthermore, Peron et al. [52] found two fluconazole-resistant *C. albicans* isolates from oral cavity (MIC=8 μg/ml) and esophageal cavity (MIC=64 μg/ml). They found six mutations encoding distinct amino acid substitutions (i.e., E116D, T128K, E266D, A298V, G448V, and G464S) that were previously reported to be associated with fluconazole resistance.

Moron and Cabrera [53] isolated 26 clinical strains from two tertiary hospitals in Metro Manila, Philippines, during November 2016 to January 2017. Out of 26 *C. albicans*, a high percentage of the isolates (73.08%) showed resistance to fluconazole. They detected the presence of point mutations T462C, A369C, and C558T. Mutations A369C and T462C have been also identified as possible factors associated with resistance to azole agents [54].

## Conclusion

In accordance with many investigations in this field, our results demonstrated that the majority of *C.* *albicans* isolates were susceptible to fluconazole. Since the main mechanism of azole resistance is the mutation occurring through the *ERG11* gene in *C. albicans*, this gene was selected for analysis in the present study. Our analysis resulted in the detection of 41 mutations in codons, only 4 of which caused changes in ERG11p. Nonetheless, these mutations (i.e., E266D and V488I) cannot lead to fluconazole resistance. Resistance among these isolates could be due to other mechanisms, such as the prevention of the intracellular accumulation of the antifungal agent and alteration of the target enzyme to diminish drug binding.

## References

[1] Naglik JR, König A, Hube B, Gaffen SL (2017). Candida albicans–epithelial interactions and induction of mucosal innate immunity. Curr Opin Microbiol.

[2] Arendrup MC, Patterson TF (2017). Multidrug-resistant Candida: epidemiology, molecular mechanisms, and treatment. J Infect Dis.

[3] Hampe IA, Friedman J, Edgerton M, Morschhäuser J (2017). An acquired mechanism of antifungal drug resistance simultaneously enables Candida albicans to escape from intrinsic host defenses. PLoS Pathog.

[4] White TC, Holleman S, Dy F, Mirels LF, Stevens DA (2002). Resistance mechanisms in clinical isolates of Candida albicans. Antimicrob Agents Chemother.

[5] Dupont S, Lemetais G, Ferreira T, Cayot P, Gervais P, Beney L (2012). Ergosterol biosynthesis: a fungal pathway for life on land?. Evolution.

[6] Xu Y, Chen L, Li C (2008). Susceptibility of clinical isolates of Candida species to fluconazole and detection of Candida albicans ERG11 mutations. J Antimicrob Chemother.

[7] Mirhendi H, Makimura K, Khoramizadeh M, Yamaguchi H (2006). A one-enzyme PCR-RFLP assay for identification of six medically important Candida species. Nihon Ishinkin Gakkai Zasshi.

[8] Yazdani MR, Foroughifar E, Mohammadi R (2016). Identification of Candida species isolated from renal transplant recipients with candiduria. Int J Organ Transplant Med.

[9] Clinical and Laboratory Standards Institute (2008). Reference method for broth di-lution antifungal susceptibility testing of yeasts.

[10] Clinical and Laboratory Standards Institute (2012). Reference method for broth dilution antifungal susceptibility testing of yeasts: fourth informational supplement M27-S4.

[11] Lai MH, Kirsch DR (1989). Nucleotide sequence of cytochrome P450 L1A1 (lanosterol 14 alpha-demethylase) from Candida albicans. Nucleic Acids Res.

[12] Löffler J, Kelly SL, Hebart H, Schumacher U, Lass-Flörl C, Einsele H (1997). Molecular analysis of cyp51 from fluconazole-resistant Candida albicans strains. FEMS Microbiol Lett.

[13] Perea S, López-Ribot JL, Kirkpatrick WR, McAtee RK, Santillán RA, Martínez M (2001). Prevalence of molecular mechanisms of resistance to azole antifungal agents in Candida albicans strains displaying high-level fluconazole resistance isolated from human immunodeficiency virus-infected patients. Antimicrob Agents Chemother.

[14] Wang Y, Wang H, Guo H, Zhao Y, Luo S (2005). Analysis of ERG11 gene mutation in Candida albicans. Di Yi Jun Yi Da Xue Xue Bao.

[15] Long F, Zhang Y, Lan H The point mutation of cytochrome P-450 lanosterol 14-? Demethylase ERG11 gene in Fluconazole-resistant Candida albicans. Chinese J Infect Dis.

[16] Li X, Brown N, Chau AS, López-Ribot JL, Ruesga MT, Quindos G (2004). Changes in susceptibility to posaconazole in clinical isolates of Candida albicans. J Antimicrob Chemother.

[17] Asai K, Tsuchimori N, Okonogi K, Perfect JR, Gotoh O, Yoshida Y (1999). Formation of azole-resistant Candida albicans by mutation of sterol 14-demethylase P450. Antimicrob Agents Chemother.

[18] Klingspor L, Tortorano AM, Peman J, Willinger B, Hamal P, Sendid B (2015). Invasive Candida infections in surgical patients in intensive care units: a prospective, multicentre survey initiated by the European Confederation of Medical Mycology (ECMM) (2006–2008). Clin Microbiol Infect.

[19] Cleveland AA, Harrison LH, Farley MM, Hollick R, Stein B, Chiller TM (2015). Declining incidence of candidemia and the shifting epidemiology of Candida resistance in two US metropolitan areas, 2008–2013: results from population-based surveillance. PLoS One.

[20] Sharifzadeh A, Khosravi AR, Shokri H, Asadi Jamnani F, Hajiabdolbaghi M, Tamami IA (2013). Oral microflora and their relation to risk factors in HIV+ patients with oropharyngeal candidiasis. J Mycol Med.

[21] Arendrup MC (2010). Epidemiology of invasive candidiasis. Curr Opin Crit Care.

[22] Poikonen E, Lyytikäinen O, Anttila VJ, Koivula I, Lumio J, Kotilainen P (2010). Secular trend in candidemia and the use of fluconazole in Finland, 2004-2007. BMC Infect Dis.

[23] Pfaller MA, Messer SA, Moet GJ, Jones RN, Castanheira M (2011). Candida bloodstream infections: comparison of species distribution and resistance to echinocandin and azole antifungal agents in Intensive Care Unit (ICU) and non-ICU settings in the SENTRY Antimicrobial Surveillance Program (2008–2009). Int J Antimicrob Agents.

[24] Pfaller M, Diekema D, Gibbs D, Newell V, Ellis D, Tullio V (2010). Results from the ARTEMIS DISK global antifungal surveillance study, 1997 to 2007: a 105-year analysis of susceptibilities of Candida species to fluconazole and voriconazole determined by CLSI standardized disk diffusion. J Clin Microbiol.

[25] Lortholary O, Renaudat C, Sitbon K, Madec Y, Denoeud-Ndam L, Wolff M (2014). Worrisome trends in incidence and mortality of candidemia in intensive care units (Paris area, 2002–2010). Intensive Care Med.

[26] Oxman DA, Chow JK, Frendl G, Hadley S, Hershkovitz S, Ireland P (2010). Candidaemia associated with decreased in vitro fluconazole susceptibility: is Candida speciation predictive of the susceptibility pattern?. J Antimicrob Chemother.

[27] Morschhäuser J (2016). The development of fluconazole resistance in Candida albicans–an example of microevolution of a fungal pathogen. J Microbiol.

[28] MacPherson S, Akache B, Weber S, De Deken X, Raymond M, Turcotte B (2005). Candida albicans zinc cluster protein Upc2p confers resistance to antifungal drugs and is an activator of ergosterol biosynthetic genes. Antimicrob Agents Chemother.

[29] Coste AT, Karababa M, Ischer F, Bille J, Sanglard D (2004). TAC1, transcriptional activator of CDR genes, is a new transcription factor involved in the regulation of Candida albicans ABC transporters CDR1 and CDR2. Eukaryot Cell.

[30] Morio F, Pagniez F, Lacroix C, Miegeville M, Le Pape P (2012). Amino acid substitutions in the Candida albicans sterol Δ5, 6-desaturase (Erg3p) confer azole resistance: characterization of two novel mutants with impaired virulence. J Antimicrob Chemother.

[31] Pfaller MA, Rhomberg PR, Messer SA, Jones RN, Castanheira M (2015). Isavuconazole, micafungin, and 8 comparator antifungal agents' susceptibility profiles for common and uncommon opportunistic fungi collected in 2013: temporal analysis of antifungal drug resistance using CLSI species-specific clinical breakpoints and proposed epidemiological cutoff values. Diagn Microbiol Infect Dis.

[32] Ying Y, Zhang J, Huang S, Liu F, Liu J, Hu X (2015). Fluconazole susceptibility of 3,056 clinical isolates of Candida species from 2005 to 2009 in a tertiary-care hospital. Indian J Med Microbiol.

[33] Berberi A, Noujeim Z, Aoun G (2015). Epidemiology of oropharyngeal candidiasis in human immunodeficiency virus/acquired immune deficiency syndrome patients and CD4+ counts. J Int Oral Health.

[34] Maebashi K, Kudoh M, Nishiyama Y, Makimura K, Kamai Y, Uchida K (2003). Proliferation of intracellular structure corresponding to reduced affinity of fluconazole for cytochrome P‐450 in two low‐susceptibility strains of Candida albicans isolated from a Japanese AIDS patient. Microbiol Immunol.

[35] Goldman GH, da Silva Ferreira ME, dos Reis Marques E, Savoldi M, Perlin D, Park S (2004). Evaluation of fluconazole resistance mechanisms in Candida albicans clinical isolates from HIV-infected patients in Brazil. Diagn Microbiol Infect Dis.

[36] Sanglard D, Ischer F, Koymans L, Bille J (1998). Amino acid substitutions in the cytochrome P-450 lanosterol 14α-demethylase (CYP51A1) from azole-resistant Candida albicans clinical isolates contribute to resistance to azole antifungal agents. Antimicrob Agents Chemother.

[37] Lamb DC, Kelly DE, Schunck WH, Shyadehi AZ, Akhtar M, Lowe DJ (1997). The mutation T315A in Candida albicans sterol 14α-demethylase causes reduced enzyme activity and fluconazole resistance through reduced affinity. J Biol Chem.

[38] Lamb DC, Kelly DE, White TC, Kelly SL (2000). The R467K amino acid substitution in Candida albicans sterol 14α-demethylase causes drug resistance through reduced affinity. Antimicrob Agents Chemother.

[39] Kakeya H, Miyazaki Y, Miyazaki H, Nyswaner K, Grimberg B, Bennett JE (2000). Genetic analysis of azole resistance in the Darlington strain of Candida albicans. Antimicrob Agents Chemother.

[40] Kamai Y, Maebashi K, Kudoh M, Makimura K, Naka W, Uchida K (2004). Characterization of mechanisms of fluconazole resistance in a Candida albicans isolate from a Japanese patient with chronic mucocutaneous candidiasis. Microbiol Immunol.

[41] Park S, Perlin DS (2005). Establishing surrogate markers for fluconazole resistance in Candida albicans. Microb Drug Resist.

[42] Maebashi K, Kudoh M, Nishiyama Y, Makimura K, Uchida K, Mori T (2002). A novel mechanism of fluconazole resistance associated with fluconazole sequestration in Candida albicans isolates from a myelofibrosis patient. Microbiol Immunol.

[43] St-Germain G, Laverdiere M, Pelletier R, Bourgault AM, Libman M, Lemieux C (2001). Prevalence and antifungal susceptibility of 442 Candida isolates from blood and other normally sterile sites: results of a 2-year (1996 to 1998) multicenter surveillance study in Quebec, Canada. J Clin Microbiol.

[44] Bedini A, Venturelli C, Mussini C, Guaraldi G, Codeluppi M, Borghi V (2006). Epidemiology of candidaemia and antifungal susceptibility patterns in an Italian tertiary-care hospital. Clin Microbiol Infect.

[45] Akbar DH, Tahawi AT (2001). Candidemia at a university hospital: epidemiology, risk factors and predictors of mortality. Ann Saudi Med.

[46] Whaley SG, Berkow EL, Rybak JM, Nishimoto AT, Barker KS, Rogers PD (2017). Azole antifungal resistance in Candida albicans and emerging non-albicans Candida species. Front Microbiol.

[47] Marichal P, Koymans L, Willemsens S, Bellens D, Verhasselt P, Luyten W (1999). Contribution of mutations in the cytochrome P450 14α-demethylase (Erg11p, Cyp51p) to azole resistance in Candida albicans. Microbiol.

[48] Teymuri M, Mamishi S, Pourakbari B, Mahmoudi S, Ashtiani MT, Sadeghi RH (2015). Investigation of ERG11 gene expression among fluconazole-resistant Candida albicans: first report from an Iranian referral paediatric hospital. Br J Biomed Sci.

[49] Eftekhari AD, Anvari M, Ranji N (2015). Investigation of ERG11 gene mutation in fluconazole resistant Candida albicans isolated from a number of Rasht hospitals. Modares J Med Sci.

[50] Alizadeh F, Khodavandi A, Zalakian S (2017). Quantitation of ergosterol content and gene expression profile of ERG11 gene in fluconazole-resistant Candida albicans. Curr Med Mycol.

[51] Balabandi S, Khazaei KZ, Ranji N (2017). Correlation between ERG11 gene mutations and fluconazole resistance in Candida albicans strains isolated from Rasht in Years 2015-2016. Arak Med Univ J.

[52] Peron IH, Reichert-Lima F, Busso-Lopes AF, Nagasako CK, Lyra L, Moretti ML (2016). Resistance surveillance in Candida albicans: a five-year antifungal susceptibility evaluation in a Brazilian University Hospital. PLoS One.

[53] Moron LS, Cabrera EC (2018). Detection of azole resistance and ERG11 point mutations in Candida albicans isolates from tertiary hospitals in the Philippines. Curr Res Environ Appl Mycol.

[54] Gołabek K, Strzelczyk JK, Owczarek A, Cuber P, Ślemp-Migiel A, Wiczkowski A (2015). Selected mechanisms of molecular resistance of Candida albicans to azole drugs. Acta Biochim Pol.

